# Advances in Laboratory Diagnosis of Coronavirus Infections in Cattle

**DOI:** 10.3390/pathogens13070524

**Published:** 2024-06-21

**Authors:** Shaun van den Hurk, Girija Regmi, Hemant K. Naikare, Binu T. Velayudhan

**Affiliations:** 1 Athens Veterinary Diagnostic Laboratory, College of Veterinary Medicine, University of Georgia, Athens, GA 30602, USA; shaun.vandenhurk@uga.edu; 2 Tifton Veterinary Diagnostic and Investigational Laboratory, College of Veterinary Medicine, University of Georgia, Tifton, GA 30602, USA; girija.regmi@uga.edu; 3 University of Minnesota Veterinary Diagnostic Laboratory, Saint Paul, MN 55108, USA; naika031@umn.edu

**Keywords:** bovine, coronavirus, veterinary, cattle, disease, diagnostics

## Abstract

Coronaviruses cause infections in humans and diverse species of animals and birds with a global distribution. Bovine coronavirus (BCoV) produces predominantly two forms of disease in cattle: a respiratory form and a gastrointestinal form. All age groups of cattle are affected by the respiratory form of coronavirus, whereas the gastroenteric form causes neonatal diarrhea or calf scours in young cattle and winter dysentery in adult cattle. The tremendous impacts of bovine respiratory disease and the associated losses are well-documented and underscore the importance of this pathogen. Beyond this, studies have demonstrated significant impacts on milk production associated with outbreaks of winter dysentery, with up to a 30% decrease in milk yield. In North America, BCoV was identified for the first time in 1972, and it continues to be a significant economic concern for the cattle industry. A number of conventional and molecular diagnostic assays are available for the detection of BCoV from clinical samples. Conventional assays for BCoV detection include virus isolation, which is challenging from clinical samples, electron microscopy, fluorescent antibody assays, and various immunoassays. Molecular tests are mainly based on nucleic acid detection and predominantly include conventional and real-time polymerase chain reaction (PCR) assays. Isothermal amplification assays and genome sequencing have gained increased interest in recent years for the detection, characterization, and identification of BCoV. It is believed that isothermal amplification assays, such as loop-mediated isothermal amplification and recombinase polymerase amplification, among others, could aid the development of barn-side point-of-care tests for BCoV. The present study reviewed the literature on coronavirus infections in cattle from the last three and a half decades and presents information mainly on the current and advancing diagnostics in addition to epidemiology, clinical presentations, and the impact of the disease on the cattle industry.

## 1. Introduction

Coronaviruses are ubiquitous in nature, and they infect a broad range of species including humans, animals, and birds. Cattle have been known to be affected by coronaviruses for many decades, with reports from as early as the mid-twentieth century [1,2,3]. Coronavirus in cattle is present in two main forms, namely a gastroenteric and a respiratory form, both belonging to the same virus in the genus *Betacoronavirus*. These are typically witnessed through three main different disease presentations according to the age and production stage of the animals [3,4,5]. The enteric form presents as neonatal diarrhea or calf scours in young animals, and older animals are described as being afflicted with the condition called winter dysentery. The respiratory form of the disease may be present in animals of all ages, but has been termed shipping fever, and older animals are typically implicated [3,5]. Considering that respiratory disease and enteric disease are often considered two of the biggest syndromes of illness and ill-thrift in cattle production systems, it stands to follow that bovine coronavirus (BCoV) is a pathogen of great importance and interest [3]. Accurate and rapid diagnostic tests for BCoV are of tremendous value, given the potential impact of the disease on animals and production. This is exemplified when considering that enteric and respiratory diseases in cattle can be caused by different infectious agents, either alone or with others as coinfections. There is still a great deal of work to be done to fully understand coronaviruses and further research may help to uncover the mechanisms that underpin the development of these varying disease presentations in cattle. It is through an enhanced understanding of viral biology, replication pathogenesis, and other factors that future research may be geared to develop more targeted diagnostic tests and intervention strategies. Rapid diagnostic testing can help a producer within a herd structure to make informed decisions regarding treatment, interventions, and preventative measures that may help to mitigate losses. Thus, it is important that our approach to the disease and diagnostics be reviewed regularly to ensure that we employ the latest technology and science at our disposal in our approach to this disease. This article seeks to review coronaviruses in cattle by including recent literature regarding the disease and diagnostics assays that may be helpful within lab or field settings alike.

## 2. The Virus

Bovine coronavirus is classified under the species *Betacoronavirus 1*, which includes a number of other viruses including coronaviruses affecting canines, pigs, horses, and others. This species is further classified under the subgenus *Embecovirus* [5,6]. The family *Coronaviridae* includes positive-sense RNA viruses containing large viral genomes of 22 to 36 kb, belonging to the suborder *Cornidovirineae* within the order *Nidovirales* [7,8]. Within this is the subfamily *Orthocoronavirinae,* which includes the four genera *Alphacoronvirus*, *Betacoronavirus*, *Gammacoronavirus*, and *Deltacoronavirus*. While the first two genera primarily infect mammals, including bats, and the last two genera primarily infect birds, mammals are also affected by some of these viruses [6,7]. Closely related betacoronaviruses include canine respiratory coronavirus (CRCoV), murine hepatitis virus (MHV), equine coronavirus (EqCoV), and the human coronavirus OC43 (HCoV-OC43), which is responsible for the common cold [2,9,10]. It is worth noting that canine respiratory coronavirus is distinct from canine coronavirus, which is an alphacoronavirus and is thus less closely related to BCoV [9]. Within these, BCoV, CRCoV, and HCoV-OC43 (as well as others) are also grouped as *Betacoronavirus 1* species viruses, and comparisons are often drawn between these agents. More broadly, the subgenus *Embecovirus* includes viruses such as MHV and human coronavirus-HKU1 (HCoV-HKU1) [2,9,11]. Other viruses in the *Betacoronavirus* genus include the severe acute respiratory syndrome coronaviruses (SARS-CoV-1 and SARS-CoV-2), which are within the *Sarbecovirus* subgenus [6,9,12,13].

**Figure 1:**
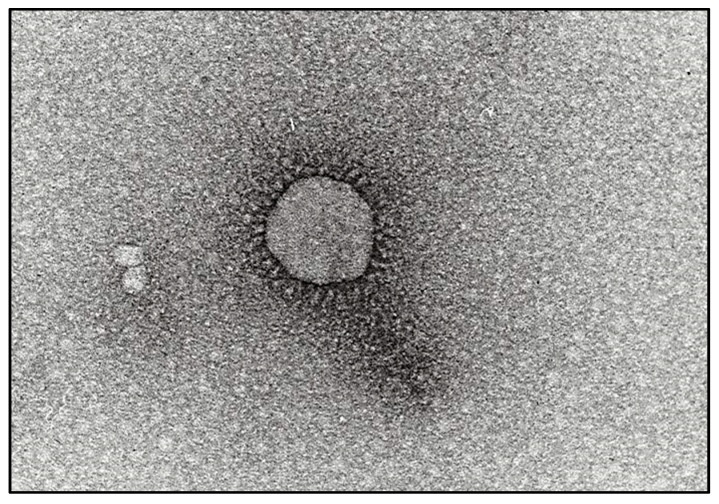
An electron micrograph (EM) of bovine coronavirus (BCoV) from a fecal sample. The characteristic halo (crown) appearance of coronaviruses from spike proteins is witnessed.

The BCoV genome is single-stranded and the 5′ end has two large open reading frames, ORF1a and ORF1b, which encode polyproteins involved in viral replication [7,14,15]. The virus makes use of ribosomal frameshifting as a mechanism to program the translation of ORF1b, following from ORF1a [7,14]. BCoVs are enveloped and pleomorphic in nature, and have five major structural proteins, namely the nucleocapsid (N) protein, integral membrane (M), Envelope protein (E), haemagglutinin-esterase (HE), and spike protein (S) [3,5]. The BCoV virion has a diameter of approximately 65–210 nm and is pleomorphic in shape [3]. The viral envelope and particularly the S proteins give the characteristic crown on electron microscopy (see Figure 1). The S and N proteins have been used in many studies as targets for diagnostic assays [16]. The spike protein is comprised of two subunits: S1 contains dominant neutralizing epitopes, and the S2 subunit mediates viral membrane fusion. While the N protein is associated with the viral RNA and found within the envelope, this is believed to be a more conserved site and thus is a good target for diagnostics [3,17]. In addition to these, there are five other ORFs that encode other accessory proteins, including the I (internal protein) gene [15].

Szczepanski et al. (2018) investigated the receptors and attachment factors of BCoV, CRCoV, and HCoV-OC43 [9]. They reported that all three viruses bound to sialic acids to an extent, but that it only served as an attachment factor for CRCoV and BCoV, and that these two viruses may use human leukocyte antigen class I (HLA-I) as an entry receptor [9]. However, the precise cellular receptor and mechanism of viral entry are still unclear, particularly how these may impact different tissue tropism or clinical outcomes. Viral entry makes use of the virion S-trimer and the host cell receptor, and it is believed that BCoV makes use of a two-receptor binding motif system [12]. Within this, Neu5,9Ac2 (sialic acid) acts as the glycan surface attachment receptor for the S1 (entry subunit) N-terminal domain [8,9,12]. The S1 C-terminal domain may bind to HLA-1 as an attachment factor that helps to initiate entry. Viral entry and membrane fusion are further initiated by the membrane-fusion subunit S2 when it is cleaved by an extracellular protease [9,12]. Hemagglutinin-esterase (HE) protein is also involved in the binding of viruses to host cells. However, HE has particularly important functions in receptor destruction and cleavage that allow newly formed virions to leave the cell [14]. These HE and S proteins are cited to play a key role in the host tropism of the virus, and thus the HE and S genes are studied for mutations and adaptations that can help to explain this tropism and virus phylogeny [1,10,12,14].

## 3. Epidemiology and Pathogenesis

There is little certainty on when the bovine coronavirus originated, but evidence suggests that it appeared around 1940 as a close relative of other *Betacoronaviruses* [1,2]. However, BCoV was first identified in the United States of America in 1972, where the virus was seen to cause severe diarrhea in cattle [18]. BCoV virus does not seem to have different variants that are responsible for the different clinical syndromes (respiratory and enteric) seen [5,19]. Rather, BCoV has been categorized into two different clusters that appear to have been caused by geographic separation [1,2]. In addition, there seems to be limited mixing of these viral subtypes between different clusters. This has largely been attributed to reduced live cattle trade between different areas, as seen with the example of Europe and the rest of the world, as many European cattle are imported from France [2]. In this way, these clusters have been largely affected and determined by major global animal trade and movement trends over the last few decades, as will be described. The two major BCoV clusters, which seem to have separated around the 1970s and 1980s, are the European and the American-Asian (North America/USA-Asian) clusters. From sequencing efforts, these clusters are seen as phylogenetically distinct, having arisen from normal viral evolution, as seen with most coronaviruses, coupled with regular selective pressures within the host population [1]. These differences have been traced back to cattle trade timelines and help indicate when certain lineages developed. Additionally, as coronaviruses are RNA viruses, regular viral evolution and mutations will account for genomic differences seen over time [13].

Taking this further, in a recent study, Bahoussi et al. (2023) described a slightly different phylogenetic grouping based on genome sequences of BCoV obtained between 1983 and 2017 [20]. This approach is similar to that outlined in an earlier study by Kin et al. (2016) [21]. Instead of the two-cluster separation with a European and America-Asian cluster, Bahoussi et al. (2023) proposed three major genogroups with subgroups within these [20]. A common feature is the strong presence of the USA and the Asian region in the maintenance and distribution of BCoV viral subpopulations and genogroups, as has been previously outlined. A divergence from the two-cluster systems previously presented is observed in genogroup 1 (G1), where we see nine different viral subtypes with the four (a to d) present in Japan and the last five (e to i) found in the USA (and potentially Canada). There is reportedly little to no mixing between these Japanese and American (USA) subtypes, indicating and supporting independent development [1,20]. Following this approach, genogroup 2 (G2) has viral subtypes largely from Europe, with France being the key region with six variants. In this approach, the oldest variants from the USA, namely the *Mebus* and *Kakegawa* variants, are also found in G2. The group G3 is mainly for a variant derived from China [20].

Another approach to the grouping of BCoV that was recommended by the same authors has the Japanese variants by themselves in genogroup 1 and all the variants from the USA, Europe, and China in genogroup 2 (with no genogroup 3) [20]. This phylogenetic grouping is interesting and provides useful information on the dynamics regarding viral evolution and recombination. It may further inform on the potential for the development of future viral variants and variants, including potential crossovers with other viruses that might possess zoonotic potential. However, when comparing the molecular epidemiology of bovine and human coronaviruses, Kin et al. (2016) indicated that BCoV seemed to have more stability than the human virus [21]. The significance of this, beyond resulting in fewer viral recombinations, is that it may help reduce the likelihood of spillover disease events to some extent [21]. As stated, evidence currently supports the idea that animal trade has determined the phylogeny of many BCoV viral variants [2]. A study by Martínez et al. (2012) indicated that, based on the S gene sequences, the BCoV sequences from Cuba were clustered along those variants from the USA, supporting a shared origin of these viruses [22]. It is believed that the USA was the source of the introduction of this virus into Cuba, although the source is uncertain. The presence of BCoV in countries in Central and South America is well documented, including the presence of BCoV-like viruses in other domestic and wild ruminants [1,12,22,23,24,25]. In Brazil, bovine coronavirus is well-established and some studies have reported positive rates between 14% and 67% in cattle [12]. It is uncertain whether the variants present originated from the USA and there is distinct variability from the USA BCoV variants. Furthermore, it has been seen that the BCoV variants present in Brazil have undergone mutations, to the extent that there are two distinct clusters when the variants are examined phylogenetically by the S protein (S1 subunit specifically) [23,26]. Interestingly, in their study, de Mira Fernandes et al. (2018) characterized BCoV variants in Brazil by the N gene. They demonstrated two distinct clusters and found some variants within these that were more closely linked to Asian variants [27]. Conversely, in Argentina, there do not seem to be multiple distinct clusters of BCoV circulating. Bok et al. (2015) did however find that the circulating BCoV variants in Argentina were still distinct from the USA Mebus strain through molecular characterization [24]. However, when interrogating these Argentinian variants for their antigenic similarity through virus neutralization, they found good cross-reactions with antibodies for the USA (Mebus) strain, indicating a single viral serotype.

The development of BCoV variants supports the notion that where there is increased localized trade at the expense of international movement, more local subtypes may be seen, sometimes with increased frequency. Meanwhile, where there is free international trade, the clusters and groups display a mix of subtypes and genomic material [2,20]. These factors may be intuitive but should be considered when attempting to control diseases or plan public health interventions. Furthermore, they should be considered for testing and developing effective vaccines for BCoV based on local variants if they are found to have significant antigenic differences.

Early field studies attempted to provide evidence that there could be a difference in viral variants leading to the different forms of disease [28]. However, some studies were conducted with a small-scale and localized population, and thus, it is possible that random minor viral mutations could have accounted for slight preferences, and that these are not necessarily representative of a larger population or long-term adaptation of the virus [1]. As mentioned, at this time there has been no report of consistent and repeatable tissue tropism leading to a particular disease presentation as caused by a specific viral variant [5,29]. There do not seem to be any changes in the spike gene that directly account for differences in tissue tropism, disease severity, or symptoms induced which would relate to the development of a specific disease syndrome [1]. However, the S1 protein, which is responsible for virus binding, does undergo changes. This, coupled with the normal mutations mentioned above, results in different variants and clusters that may be seen over time and geographically [5,29]. This is witnessed through the fact that modern viral variants differ far more from older viral variants than those collected from different clinical syndromes [5,29]. The role of different viral molecular factors in tissue tropism and disease outcome is still being investigated [30]. Multiple studies have used viral isolates from one clinical form of BCoV, i.e., winter dysentery, and induced respiratory disease when inoculating or exposing experimental hosts with this same viral isolate (and thus variant) [31,32]. Additionally, studies have demonstrated the shedding of BCoV from both respiratory and fecal routes from the same animals, thus further supporting a lack of specific tissue tropism [33,34]. Thomas et al. (2006) and Zhang et al. (2007) identified and proposed the existence of a BCoV quasispecies, with intrahost viral variation and evolution, and this could help to account for some of the observed differences between viral isolates and predominance of certain clinical signs as witnessed [35,36]. Further studies are indicated to help elucidate the underlying deterministic factors when considering the development of different clinical syndromes from BCoV infection in cattle.

Various domestic and wild ruminants have been seen to be affected by bovine-like coronaviruses that display close similarities to BCoV. It is believed that these viruses may be novel host-range variants of the BCoV virus that could occur through contact of cattle with these species, such as bubaline coronavirus (BuCoV) in water buffalo [37]. HM Amer (2018) indicated a very high degree of sequence identity between BCoV and BuCoV with genome sequencing (98 to 99%) and indicated key and marked differences in accessory proteins between the S and E protein regions [37]. Furthermore, phylogenetic tree analyses were provided based on the N and S genes that help to illustrate the close relationships between these BCoV-like viruses and variants in domestic and wild ruminants [37].

## 4. Clinical Presentations

Bovine coronaviruses affect the respiratory and gastrointestinal tracts of cattle and can cause mild to severe disease. While disease outbreaks on a farm may typically present in one distinct form, this is not always the case and BCoV might cause any (or all) of the three main clinical syndromes [5,19,31]. In their study, Oma et al. (2016) had calves that developed respiratory disease after being exposed to cows with typical winter dysentery diarrhea [32]. Additionally, it has been noted that cattle may shed the virus from both the enteric and respiratory routes at the same time upon infection [34]. The shedding of the virus is not only important for direct transmission through contact but also for delayed transmission (and in some cases sustained presence) as coronaviruses are seen to be relatively resistant despite being enveloped [25]. The pathogenesis and clinical signs of the respiratory and enteric forms of the disease are presented separately below for ease and in keeping with much of the available literature, which often investigated and presented these two as different syndromes.

### 4.1. Respiratory Syndrome

Where infection takes place in the respiratory system, the virus initially replicates in situ within the nasal turbinates, trachea, and lung epithelial tissue. The virus primarily affects the epithelial cells of the respiratory tract, with attenuation and necrosis seen in these cells microscopically. Most commonly, tracheitis is seen along with rhinitis and interstitial pneumonia [5,38]. The most common clinical signs seen are coughing and rhinitis which may display with nasal discharge (serous to mucopurulent), and this may be coupled with pyrexia and anorexia. This can further progress to include dyspnea, tachypnea, and pneumonia, with open-mouth breathing and wheezing being observed in animals, and death may occur in severe outbreaks [5,19,29]. All animals and age groups may be affected by the respiratory syndrome, and there is no distinct difference in the way that different animals are affected by this, which differs from the gastrointestinal syndrome. Younger animals (calves between 2 and 9 months of age) have historically been highly affected by BCoV infection, with clinical signs being seen less frequently in older animals [19,29,39]. However, one should consider whether this is affected by the fact that cattle typically enter beef feedlot settings around 6 months of age, and this presents a very high-risk event for infection and disease transmission, especially those related to bovine respiratory disease complex (BRDC). Multiple studies demonstrated the high prevalence of BCoV in beef feedlots and even dairy systems.

While investigating risk factors related to outbreaks of acute respiratory disease in calves of mixed origin (beef and dairy) in Belgium, Pardon et al. (2020) frequently found BCoV, with viral presence in 38.4% of outbreaks by PCR (polymerase chain reaction) detection [40]. When evaluating BCoV shedding in feedlot calves, Thomas et al. (2006) detected BCoV being shed in 73% of calves through nasal and fecal shedding before arrival at a feedlot [36]. Zhang et al. (2021) demonstrated a high prevalence of BCoV using nanopore-based metagenomic sequencing of deep nasal swabs in cattle upon arrival at a feedlot in Western Canada [41]. In this study, BCoV was the viral agent identified with the highest prevalence at 45.2% of the cattle sampled. Interestingly, when these animals were monitored for 40 days, it was noticed that there were near equal numbers of cattle that had BCoV identified in their nasal virome and either remained healthy or displayed these BRD signs [41]. Similar results were seen in a study performed in dairy cattle herds in Brazil where BCoV had the highest detection rate of all pathogens detected at 56% [30]. In this study, the researchers tested calves from 5 to 90 days of age using PCR and molecular characterization through gene sequencing of N and S1 genes. Even in this instance, there was little difference in the detection rates of BCoV between those calves displaying clinical signs and animals that did not show signs. It was suggested that this could be affected by potential early cases, persistent and/or subclinical infections within these calves [30]. It has been shown that cattle that shed the BCoV virus are more than twice (2.2 times) more likely to have pulmonary lesions at slaughter, and that animals that shed virus and have an antibody response to BCoV are 1.6 times more likely to require treatment within a feedlot setting [42].

The impact and precise role of BCoV as a respiratory pathogen in the BRDC is still debated by some. However, it is clear that high detection rates of the virus have been noted in cattle with respiratory disease, either alone or with other pathogens and the virus has caused pathology through inoculation and infection in other studies [5,29,31,32,39,40,41,43]. More specifically, further studies have demonstrated the presence of the virus in respiratory tracts with histological evidence of pathology [38]. In this study, immunohistochemistry and an RNA in situ hybridization assay (ISHA) were both used to identify the presence of BCoV in the lower respiratory tract (the focus on the trachea and lungs) of calves. A substantial number of the tracheal tissues evaluated demonstrated epithelial attenuation in conjunction with the presence of the virus as seen through the IHC and ISHA tests. However, the authors noted that it is hard to directly attribute the pathology identified solely to BCoV, as in most cases there was coinfection with other viral and bacterial pathogens that form part of the BRDC. However, these samples were derived from cases that succumbed to disease and thus may have progressed from primary viral to secondary bacterial infections, which could further impact the isolation of pathogens. In the study, the virus was demonstrated in cells with clear microscopic evidence of epithelial damage [38].

### 4.2. Gastrointestinal Syndrome

While the respiratory form of the disease may affect all animals with little preference or difference in observed symptoms, the gastrointestinal forms of the disease can be categorized according to whether they affect calves or adult cattle [3,5,19]. In calves, we see a neonatal diarrhea syndrome, which typically affects animals within the first three weeks of life. This diarrhea can vary in character from watery to mucoid and pasty, either with or without blood. Calves are primarily affected by malabsorptive diarrhea caused by necrotic enteritis, which may be present throughout the intestinal system. This typically starts in the small intestine around the duodenum and settles in the colon with villous atrophy and increased crypt length in the small and large intestines, respectively [5,19,31]. It is further possible to see hemorrhagic diarrhea in some calves. The lesions and disease presentation within the gastrointestinal form will further be affected by the route of infection. The incubation period in calves is observed as three to seven days, with the duration of clinical symptoms lasting three to six days, although this may also be affected by the route of infection [3,5,19]. The mortality rate is typically low, but this is variable and will be affected by animal factors such as age and immune status.

In adult cattle, the disease form seen typically is called winter dysentery. Historically this was reported as a self-limiting form of diarrheal disease affecting all adult animals, with a particular predisposition in postpartum dairy cows. However, severe infections in adult cattle have also been seen, including outbreaks of disease outside of the cold or winter season, which was historically considered to be normal [43]. As with most infectious agents, immunologically naïve or compromised animals are most likely to display symptoms of the disease and increased viral shedding upon exposure or inoculation. This is directly seen with parturient and postpartum cattle that shed BCoV at higher levels [3]. As with calf diarrhea caused by BCoV, the pathological lesions present in adults include villous atrophy and increased crypt length in the intestinal system. However, in adult animals, watery diarrhea is the most common observation with hemorrhagic diarrhea possible in some cases [5,19,43,44]. The main deleterious effect of winter dysentery in adult cattle can be ascribed to the production losses encountered, especially in dairy cattle in lactation [45]. Loss of production from BCoV infection can be immense, particularly when considering that this is typically a disease that will spread quickly in a herd and, if there is no quick intervention, may affect a large number of animals from all age groups.

The complex interplay and presentation of the disease forms are reinforced by two separate studies where calves were inoculated and infected with the enteric form of the disease and developed respiratory disease and pathology. Park et al. (2007) inoculated calves orally with a Korean winter dysentery BCoV variant, after which the calves developed severe pathological lesions in the gastrointestinal and respiratory tracts [31]. The upper and lower respiratory tracts were affected with epithelial damage in the nasal turbinates, trachea, and lungs along with interstitial pneumonia in the calves. The BCoV viral antigen was found in the epithelium of all these affected tissues. In this study, the symptoms and pathology first developed in the gastrointestinal tract (villous atrophy and increased crypt depth in small and large intestines), and thereafter the pathology developed in the respiratory tract tissues [31].

A further experimental field study was performed by Oma et al. (2016) to help understand the viral shedding and clinical effects of BCoV in calves that were naturally infected (not inoculated with the virus) [32]. The study found an association between viral shedding (nasal and fecal) and clinical signs, especially in the acute stages of infection. Interestingly, shedding of BCoV viral RNA was seen to persist for five weeks in these naturally infected calves, although they were only seen to be infectious for three weeks from exposure [32]. However, as has been discussed, viral shedding is not linear and may not be directly dependent on or correlated with clinical signs. Within the course of infection, animals are typically seen to shed viral particles from both the fecal and nasal routes even in cases where no symptoms affecting the other system might be noted [32]. It is believed that there might be a higher limit of detection in fecal samples (reduced sensitivity) compared to nasal samples, which can affect findings, and thereby this favors nasal samples for the detection of viruses. In agreement with the findings of Park and colleagues, it is further suggested that the initial shedding of BCoV in calves might be affected by the primary route of infection with the virus [31,32]. Where infection takes place through the respiratory route, which may be more common in field settings, viral replication and shedding will first occur in the respiratory tract, with nasal shedding seen. If viral infection or inoculation takes place through the oral route (experimentally or through indirect exposure), then viral replication will initially occur in the gastrointestinal tract, with fecal shedding occurring first [32,36].

In the Oma et al. (2016) study previously cited, the calves infected in the field were exposed to an outbreak of winter dysentery affecting adult cattle in the field, but once the calves were infected the predominant syndrome and clinical signs experienced were that of respiratory infection [32]. This is interesting as early investigations and many studies have sought to evaluate how different syndromes of respiratory and gastrointestinal disease can exist in animals infected with BCoV. As stated, evidence suggests that differences in how the virus is spread and enters the host might affect initial tissue tropism and consequent viral shedding, but importantly, the same infectious agent underpins both of these processes independent of the clinical signs [5,19,29,36].

The response to BCoV infection and consequent shedding appears to be very variable, but there is evidence to suggest that increased antibody levels at the time of exposure or inoculation are related to decreased shedding of the virus through nasal and fecal routes [36]. This is important to note when interpreting findings from studies that investigate and report viral shedding of BCoV in cattle. Furthermore, there is a practical application to this, as vaccination of cattle before high-risk events such as transport, exposure, and comingling of new animals could help to reduce viral shedding and thus disease incidence in these animals [36]. Determining the full impact of BCoV on the health and production of cattle is challenging due to the multifactorial nature of the BRDC and diarrheal diseases. It can be a challenge to quantify the additive effects of the environment, stress, and other pathogenic agents that may be present in disease syndromes. Additionally, a lack of consistency in replicating the clinical effects of BCoV infection in experimental studies only serves to complicate this further [36]. However, several studies have demonstrated decreased growth performance in cattle infected with BCoV, especially in the feedlot setting [33,34,36,46]. Further supporting the recommendation for immunization through vaccination, early studies have demonstrated that increased weight gain of feedlot calves is witnessed in those with increased antibodies to bovine coronavirus [29,46]. Even though it might be hard to ascertain the exact effects of BCoV, there is a substantial degree of evidence to indicate that the presence of the virus is linked to increased risk for clinical disease and the presence of pathological lesions, with consequent decreased production [32,33,34,42].

## 5. General Aspects of Diagnosis

Many factors affect the accurate diagnosis of a disease or identification of a causative agent. This starts with the infected host, relevant sample collection techniques, specimen handling, and transport, and lastly, the diagnostic tests performed. First, the diagnostic assay should be the most appropriate test for the pathogen, immune response, disease process, and herd situation. This is seen primarily when deciding between the identification (and in some cases quantification) of a pathogen (antigen) or the presence of a pathogen versus the identification and quantification of an immune response (antibodies) against this pathogen. It is critical to understand the inherent advantages and disadvantages of different diagnostic assays and to ensure that the interpretation of the results considers these factors. In this regard, the main diagnostic assays that are currently available for BCoV (Table 1) are nucleic acid-based tests (notably Polymerase Chain Reaction (PCR)), Enzyme-Linked Immunosorbent Assay (ELISA), viral neutralization test (VNT), immunohistochemistry, fluorescent antibody tests, and electron microscopy. The development of point-of-care tests (POCTs) that follow the principles of some of the diagnostic methods mentioned above is ongoing.

Sample collection for the diagnosis of BCoV infection should consider the disease progression and tests to be performed, amongst other factors. Given that BCoV has enteric and respiratory forms of disease, the collection of samples for viral identification should ideally match the disease state witnessed. However, when considering serological tests, the collection of samples will be independent of the form of disease, as the collection of a blood (or milk) sample is the current standard. A thoughtful interpretation of results from serological tests is needed as BCoV may be included in routine vaccination protocols, and antibodies may be present in apparently healthy animals. Lastly, in the case of deceased animals, judicious collection of diagnostic specimens at post-mortem will greatly enhance diagnostic value, and consultation with a veterinarian or pathologist is advisable before collecting specimens for testing.

In the enteric form of BCoV infection, a fresh fecal sample may be used for pathogen identification tests such as PCR. It should be kept in mind that fecal samples may have inhibitors present that can interfere with nucleic acid based-diagnostic tests and that this might influence test results [29,64]. In the case of BCoV respiratory syndrome, a nasal swab (NS), nasopharyngeal swab (NPS), bronchoalveolar lavage (BAL), or transtracheal aspirate (TTA) are appropriate sample types for pathogen detection. A study conducted in cattle to compare these different approaches identified that not all approaches might be equally efficacious and suited for accurate identification of all respiratory pathogens [11]. Some pathogens in the bovine respiratory disease complex, especially bacterial pathogens such as *Mannheimia haemolytica* and *Pasteurella multocida*, might have better agreement between different sampling methods such as NS, NSP, and TTW. On the contrary, some of the viral pathogens might have a weaker correlation between these testing methods, which was seen to be the case for BCoV. There was a significant difference between the commonly used upper airway collection methods of NS and NSP, and even the lower airway collection of BAL, compared to TTW, which might call into question how well-suited all of these methods are for diagnostic sample collection [11]. In this case, it was seen that more animals tested positive from all three other methods (NS, NSP, and BAL) than TTW [11]. Additional consideration should be given to the effect of nasal shedding of the virus from the upper airway and nose in infected cattle, and also to the fact that BCoV can often be isolated from the upper airways of apparently healthy cattle [30,42,69]. Additional studies are warranted to evaluate this further and to ascertain the best approach to sample collection for each respiratory pathogen.

When it comes to nucleic acid-based diagnostic assays for the identification of pathogens, FTA^®^ cards provide an efficient means to store and transport sampled materials. FTA^®^ cards are chemically treated and enhanced filter paper cards that are used to extract and store nucleic acid from an applied biological specimen for further testing at a later time [70]. This is a direct alternative to the collection of samples with subsequent storage and transport in specialized transport media and controlled environmental conditions, as the FTA^®^ cards are environmentally stable. These cards have been shown to be effective and reliable for nucleic acid identification for a wide range of pathogens and collected samples [70]. This is very beneficial for diagnostics in cattle production systems, where field and testing conditions might be far from diagnostic services and where it might not be feasible to maintain ideal storage and transport conditions for samples. Thus, the FTA^®^ cards would provide a possible option for the collection of samples from cattle where BCoV is suspected, whether this is the enteric or respiratory form. Liang et al. (2014) investigated the use of FTA cards for specimen collection and PCR testing of viruses associated with the BRDC [71]. In their study, it was shown that the cards presented a viable and favorable alternative to viral transport media when used for nasal swabs collected from cattle and tested by real-time PCR for bovine respiratory syncytial virus (BRSV), BCoV, bovine herpesvirus type 1 (BHV-1) and bovine viral diarrhea virus (BVDV). It should be kept in mind that FTA^®^ cards are primarily used for the identification of pathogens through the presence of nucleic acid, and thus, this is helpful for qualitative tests such as PCR and gene sequencing. However, these may not help quantify the viral load and should be interpreted with caution in situations where the pathogen can be found in healthy animals. Thus, pairing these tests with serological tests may be advantageous to help track a potential disease outbreak. Additionally, the use of a real-time PCR from fresh samples could be useful in situations where quantitative measurements are desired. Furthermore, for modalities such as gene sequencing, higher amounts of nucleic material may be required than can be obtained through these cards, and this may affect the choice of sample collected.

If presented with a dead animal, tissue samples from the digestive and respiratory tracts form the basis of diagnostic samples to be collected at necropsy. This will include samples from the upper and lower airways, with emphasis on the nasopharyngeal tissue (nasal turbinates and pharynx), the bronchus, and the alveolar tissue from the lung to help assure sufficient coverage [19,29,31,38,72]. The gastrointestinal tissues should focus on the intestinal tissue, such as the ileum and the colon, trying to ensure sufficient tissue sampling and reducing excessive fecal contamination where possible. In general, it is a good practice to include regional lymph nodes when submitting samples of tissues wherever possible [19,32].

It is important to remember that diagnosis for BCoV infection is best performed early, especially where pathogen identification and quantification are desired. This is because BCoV typically presents as an acute disease with peak viral shedding found in the first 3 to 5 days of infection [29,33,73]. Considering the usual lag between symptom onset and management intervention, this might result in a limited window to catch the peak shedding of the virus. In a typical infection, there may still be shedding of the virus for up to three to five weeks, although this has not been seen as infectious at this point and is unsuited to viral isolation (propagation) [32]. Typically, after the first five days, the virus may be shed at decreasing levels with time or may be shed intermittently, making diagnosis challenging.

In addition to gastroenteric and respiratory samples, conjunctival swabs have been used to identify BCoV viral shedding in some studies [43,49]. The utility of this for diagnostics at the field level will need to be identified, but this may provide a good additional option for sample collection for potential cases where fecal or nasal contamination reduces diagnostic sensitivity. Further supporting the early collection of samples is the fact that some infected animals may experience viremia (often coinciding with periods of pyrexia), and blood samples collected at this time can be used in pathogen detection [19,31,32]. Additionally, the use of blood (plasma and serum) and milk samples can be of great value to help demonstrate exposure to the virus through the presence of antibodies, and this is of even greater utility when quantification of the response is possible. There will always be a lag in the response for all classes of antibodies, which means that levels may not increase to detectable levels until a few days post-infection. Typically, increases in the IgA and IgM antibody levels can be used as an indication of a recent exposure, whereas increases in IgG antibody levels (with the exclusion of IgA and IgM) will indicate exposure in the past [74]. However, in veterinary practice, many assays will not be able to distinguish between these different antibody classes. Therefore, the collection of paired serum samples (two samples two to four weeks apart) is usually recommended in live animals to help establish and demonstrate more accurately when potential exposure might have taken place [74]. This is particularly pertinent with a disease such as BCoV where there is a very high prevalence of the disease and when the clinical history of animals is unknown, such as upon arrival at farms from an auction. This can make diagnosis with serology challenging and add many variables. As a rough guide, a significant increase in serum antibody levels following arrival at the farm will indicate recent exposure and infection, while a high antibody level at arrival that wanes thereafter may indicate prior exposure [73]. This should be coupled with an interpretation of the relative amounts of the different antibodies as stated. The limitation of the collection of paired serum samples approach is the time that elapses between sample collections. This can be overcome by using assays that can quantify the response and provide a result compared to a baseline, rather than a purely qualitative test, which is helpful for acute diagnosis. An excellent review on the use of serology as a diagnostic assay for infectious diseases in bovines was recently published which covered many of the important considerations when utilizing these diagnostic assays, and is cited [74].

Molecular tests that focus on nucleic acid detection, in particular PCR and its derivatives, have become the gold standard for routine diagnostics and viral detection [29]. This is due to the very high sensitivity and specificity that is offered by PCRs. These tests are usually more rapid to perform and do not require the same level of technical expertise as electron microscopy. As a negative-strand RNA virus, BCoV necessitates an RT-PCR (reverse-transcription PCR), and further tests in this line include qRT-PCR (quantitative or real-time PCR) and nested-PCRs [5]. These expanded nucleic acid assays are valuable as they offer the ability to help quantify viral load and thus shedding, which can help with further interpretation of results. In a similar line to PCR tests are microarray assays, isothermal amplification assays including RPA (recombinase polymerase amplification), LAMP assays (loop-mediated isothermal amplification), HDA (helicase dependent amplification), and MIRA (multienzyme isothermal rapid amplification)-based assays, among others. These assays typically offer high levels of sensitivity and specificity. Another potential diagnostic assay that has grown in popularity and use recently is genome sequencing, which also has high levels of sensitivity and specificity. The main limitations of genome sequencing as a diagnostic tool currently are the cost of testing per sample in the absence of high volumes of samples to offset the price, and the need for expensive instrumentation. Additionally, samples need to be of a high quality (sufficient and good quality nucleic acid to test) and ideally isolated without host DNA. As such, the main diagnostic use of genome sequencing at this time is for the characterization of pathogens rather than for detection alone.

Several serological assays, including Enzyme-linked immunosorbent assays (ELISAs), are commonly used for the laboratory diagnosis of BCoV infections. This can take two different forms: detection of viral antigens, or detection of antibodies to the virus (immune response and thus exposure) [29,75]. A further development of this is immunochromatography tests (ICTs), which often include lateral flow, and make use of similar principles to that of ELISA to offer rapid and typically on-site assays. In addition to ELISAs, other tests to help identify a serological response to BCoV infection include VNT and hemagglutination inhibition (HI) tests [29]. Virus neutralization tests may still be used to help quantify an antibody response and thus inform on infection and immunity [76].

Traditionally, many laboratory diagnostic tests require the use of specialized equipment and highly skilled personnel, which makes it necessary to send samples to well-funded and highly equipped diagnostic laboratories. As time and technology have evolved, this has changed, and in essence, many diagnostic assays have become more accessible, with many local clinics and satellite laboratories being able to run these assays. However, even within these, in some cases, there is still a need for costly and sophisticated equipment that requires ongoing maintenance and is not portable. Therefore, new diagnostic technology is looking towards point-of-care tests (POCTs), which can offer reliable diagnostic tests on-site or barn-side, on the farm [77]. Point-of-care tests have been available in different forms for some time in veterinary medicine, especially for use in companion animals. These assays have allowed veterinarians to screen for diseases at the time of consultation to provide an initial diagnosis, and if further validation is required then a secondary diagnostic assay may be used. The advantages to this approach are immense as it allows for rapid decision-making making, facilitating early management and treatment actions, in turn providing better responses to treatment and reduced costs. There are a number of POCTs available for pathogens affecting bovines, many of which were traditionally based on ELISA and ICT. However, nucleic acid-based tests have become more common, and isothermal amplification assays will further enhance this development. Many POCTs make use of microfluidic systems in their operation and may use lateral flow to facilitate the read-out of results [77]. Digital technology and artificial intelligence are more available than ever, and there is a shift to the integration of these within POCTs for in-field veterinary use. Further development of and investment in POCTs for use in livestock disease diagnostics will greatly support veterinarians and producers alike, as this will inevitably make these assays more accessible and affordable and thus enhance welfare and production.

## 6. Laboratory Diagnostic Assays

### 6.1. Virus Isolation

It is possible to culture BCoV where viral isolation (and plaque assay) is desired, and typically HRT-18 (Human Rectal Tumor-18) cell lines are used for this. However, various other options for potential cell lines for culture exist, such as using human adenocarcinoma (HCT-8), bovine embryonic lung or kidney cells, or bovine spleen cell lines, to name a few, each with their own advantages and disadvantages [78]. It is challenging to culture the virus from clinical samples, and this may affect the overall sensitivity of the tests [4,29,75,79,80]. Additional viral detection assays may include immunofluorescent or immunohistochemical staining with tissue samples. For these techniques, monoclonal antibodies or hyperimmune serum against BCoV may also be used [50].

### 6.2. Electron Microscopy

One of the initial diagnostic tests for BCoV was based upon the physical detection and identification of the virus through electron microscopy (EM) [4,81,82]. Electron microscopy can be used for virus identification from any samples with virus present (traditionally fecal and nasal samples) [4,29]. Overall, EM offers moderate specificity, although this specificity increases at a family level. However, the sensitivity of EM is low (especially compared to molecular assays) and is typically more time-consuming than most antibody or nucleic acid tests, thus making it less favorable in the current diagnostic setting. Additionally, EM requires highly skilled and experienced personnel, as well as expensive specialized equipment that takes up a lot of physical space. For these reasons, EM is no longer routinely used for the diagnosis of BCoV in favor of more modern techniques that are easier to run and have higher sensitivity and specificity. Virus detection has largely moved beyond physical identification based upon gross structure to molecular tests that use sophisticated techniques to detect specific markers and other molecular characteristics for reliable detection and identification of viruses down to a specific variant or subtype.

### 6.3. Polymerase Chain Reaction (PCR)

In terms of the detection of BCoV, PCR is the gold standard assay and the most widely used assay for diagnostic and research purposes [44,48,49]. Traditionally, these PCR tests have been based on single detection with specific primers and markers used to facilitate and enhance the detection of the pathogen [16,17,47,48]. An advantage of all PCR tests is that they provide high levels of sensitivity and specificity [16,17,47,48,49]. This is provided with the use of well-designed primer sequences specific for a particular target site in the organism’s genome to facilitate selective and precise annealing of these nucleotides, followed by amplification and detection. Further development of PCR assays has enhanced the sensitivity and specificity even further, and in the case of real-time (quantitative) PCRs, the use of specific probes labeled with fluorescent dyes helps to report and quantify the presence of the pathogen in real-time [48,49].

The two main methods that are used for this fluorescence are SYBR Green and TaqMan. The specificity of this fluorescence will be determined by the markers used, as they have differences in their binding mechanism and specificity. In essence, TaqMan can be seen as more sensitive and specific for the diagnosis and detection of BCoVs [16,48,49]. The degree of fluorescence will give an indication of the viral load (quantity of nucleic acid or targets) beyond simply providing evidence of the target pathogen’s presence, which is illustrated during the test procedure (real-time). In this way, a PCR assay is able to provide a quantitative (or semiquantitative) aspect to what was historically a qualitative assay and provides a more rapid and sensitive result. Thus, quantitative PCRs help to increase the sensitivity of the assay compared to direct PCR without that component [16,49]. Nested and semi-nested PCRs also provide a further means to improve the sensitivity of the diagnostic assay, as the two-step process focused on target sites helps to reduce the occurrence of false negatives and false signaling when used with fluorescence [50].

The use of nested PCRs was introduced to help increase diagnostic sensitivity, as reactions and thus detection are derived through two stages of binding and amplification from the designed primers; usually, the second primer has a smaller sequence size than the first primer or is a more highly conserved region. With the general improvement of PCR primers and reactions, along with multiplex PCRs (some of which follow some principles from nested PCRs) and other genome and proteome-based assays, nested PCRs in isolation are not common. However, they do offer a practical and effective method to improve the sensitivity of assays, and researchers have demonstrated their effectiveness for BCoV. In three studies reviewed, all three made use of the N gene of BCoV as a target for the primers used and all three noted increased sensitivity of the assays compared to a one-step PCR, with up to a 100-fold improvement in sensitivity [50,51,52]. Furthermore, Takiuchi and colleagues (2006) reported that the semi-nested PCR assay was able to detect BCoV in fresh and frozen fecal samples, while the conventional PCR test could only detect BCoV in the fresh feces, once again highlighting the increased sensitivity of this approach [51].

Other factors that may affect the diagnostic sensitivity include the diluent and other media used in the assay. Asano et al. (2009) proposed that the amount of total RNA in the fluid helped to improve the sensitivity of the test [50]. This is likely because the higher RNA concentration assisted the formation of a good pellet and potentially helped carry the RNA in extractions to reach the target for the assay [50]. A disadvantage to PCR and other similar assays that target specific proteins or genome structures is that primers have to be made based on this section of the viral genome, and thus, the pathogen has to be known. Furthermore, if the virus undergoes significant changes or mutations affecting this targeted site, there may be a reduction in the sensitivity and specificity of the test as the assay no longer reacts with the structure. Thus, assays are typically designed to target highly conserved regions of pathogens. The nucleocapsid (N) gene of BCoV is used as a good target for PCR assays as it is well conserved among variants and thus improves the sensitivity of the assay [17,47,50,83]. Escutenaire and colleagues (2007) made use of the BCoV ORF1b region in their RT-PCR assay, citing this as a more conserved site than the N gene and thus better for detecting viruses from the different clusters [48].

### 6.4. Multiplex PCR

Traditionally, assays based on PCR or qPCR for a single pathogen or target (singleplex) have been the mainstay. This is because they are intuitively easier to design and perform, as reagents and reactions are more focused. However, the ability to detect multiple pathogens at the same time through a single test procedure, such as duplex, triplex, or multiplex assays, is advantageous and attractive. If a single assay is able to detect multiple pathogens at the same time, it helps to save sample volume, costs on reagents and materials, saves time, and helps to streamline the diagnostic process and facilitates screening. It is for these reasons that multiplex PCRs are becoming more widespread, especially in diagnostic settings. These assays can differ based on the number of pathogens that are targeted by the assay as well as the technology that is used to facilitate reading and interpretation of results, but largely they follow the same approach as traditional PCRs. The pathogens targeted can be from the same viral family or based upon syndromes with unrelated agents to provide a diagnostic panel for a particular disease syndrome (e.g., gastrointestinal or respiratory disease). It is convenient for a producer or clinician to have a single diagnostic panel that tests for differential diagnoses related to a clinical sign.

An assay designed to screen for different viruses within a family is witnessed in a study by Loa et al. (2006), who developed a multiplex PCR for the detection of turkey coronavirus (TCoV), infectious bronchitis coronavirus (IBV), and BCoV [55]. The researchers made use of primers targeting the N and S genes of the viruses (the S gene was used for BCoV) within the same reaction and reported high sensitivity and specificity for these agents with their assay. Similarly, another triplex qPCR was developed by scientists in China for the detection of bovine parvovirus (BPV), BCoV, and bovine parainfluenza virus (BPIV), all of which are responsible for gastrointestinal and respiratory disease in cattle [54]. The triplex PCR yielded a diagnostic sensitivity 1000 times greater than the conventional PCR, high sensitivity with a limit of detection of 2.0 × 10^2^ RNA copies/μL, and improved efficiency in clinical samples [54].

As an important pathogenic agent responsible for diarrhea and gastrointestinal disease in cattle, BCoV has been included in a number of studies that developed multiplex PCRs targeting enteric pathogens in calves and adult cattle [53,54,84,85,86,87,88]. Furthermore, BCoV is also an important agent in the BRDC and as such is also included in multiplex PCRs and screens targeting respiratory pathogens [54,76,89]. Therefore, there is great interest in developing effective assays that can screen and detect BCoV along with a number of other respiratory and gastrointestinal pathogens in order to save resources and assist producers and veterinarians. Cho et al. (2010) developed a multiplex real-time PCR assay to detect five enteric pathogens (BCoV, BRV, *Salmonella* spp., *E. coli* K99^+^, and *Cryptosporidium parvum*) and tested this assay on fecal samples with known infection status [84]. The assay was validated against traditional tests for each pathogen and reportedly outperformed these assays, as it was seen to be more sensitive and rapid than the traditional tests. The authors reported an agreement of 89–97% between the conventional tests and the multiplex assay, and stated that in many cases the incongruency was attributed to the higher sensitivity of the multiplex PCR panel [84]. Within their study, the authors reported that they were able to optimize the PCR extraction method to save time and also to minimize inhibition from fecal samples. The total time to run the full panel for 96 samples was around 4 h, with 1.5 h for sample extraction and 2.5 h for the PCR [84]. This highlights the benefit of these multiplex assays that can screen large numbers of animals for a few pathogens of interest at the same time with the same level of inputs. A similar study was published more recently by Pedroso et al. (2023), where the researchers developed an end-point multiplex PCR/RT-PCR for the same five enteric pathogens as the previous study [53]. The authors ran singleplex PCRs to compare to the multiplex and indicated that the multiplex assay also provided very high sensitivity and that the detection limit for BCoV was a 10^−2^ dilution of the positive sample pool. The specificity of the assay was high as there were no cross-reactions between the pathogens and no reaction to other agents known to cause enteric disease [53]. These multiplex assays are of great value when faced with a disease syndrome such as diarrhea or respiratory disease where multiple agents may be causative, or additive, and swift diagnosis and intervention is desired.

Multiplex qPCRs aimed at pathogens responsible for respiratory disease in bovines were investigated by researchers in Denmark and Japan [76,89]. The research group of Pansri et al. (2020) evaluated two commercial multiplex qPCR kits, one for four bacteria (Pneumo4B—Diagnostic A/S, Risskov, Denmark) and one for five viruses (Pneumo4V—Diagnostic A/S, Risskov, Denmark), the latter targeting BPI3, BCoV, BRSV, bovine herpes virus-1 (BHV-1), and bovine viral diarrhea virus (BVDV) [89]. The assays tested performed well, as they were seen to outperform the reference singleplex qPCR assay. For BCoV. the sensitivity was 100%, the specificity was 95% with a kappa value of 0.90, and the reported limit of detection was 10 RNA copies per reaction [89]. Goto and colleagues (2023) investigated the use of a multiplex qPCR on nasal swabs as a means to detect twelve bovine respiratory disease pathogens (eight viruses, including BCoV, and four bacteria) and questioned the utility of this to monitor for infection [76]. They sampled clinically healthy and infected calves, and in the infected calves, they performed viral neutralization tests (VNTs) to elucidate whether these animals had an immune response that confirmed infection. The researchers indicated that there was a close relation between positive VNTs (increased antibody titers) and positive multiplex qPCR results, which supported the fact that the PCR is well suited to monitoring for infection [76]. There was no singleplex PCR used as a reference assay but the use of the field samples and VNTs helps to support the utility of this assay in practical settings. These studies clearly demonstrate the value of multiplex PCR assays and support their widespread use in practice, especially since they are seen to provide increased sensitivity, as well as reduce time and costs related to the diagnostic procedure.

### 6.5. Droplet Digital PCR (ddPCR)

Droplet digital PCR provides another nucleic acid-based assay that can detect viral particles but does not involve Ct values or standard curves, which can help yield increased sensitivity of the assay. Researchers in China developed a ddPCR assay that was able to concurrently detect multiple agents involved in BVDV (BCoV, bovine rotavirus, and bovine enterovirus) and tested and validated the assay on clinical samples from three different farms [90]. The authors found that the sensitivity of the ddPCR was up to 1000 times more than their qPCR, able to detect BCoV at 1 copy/μL, and that the assay could easily differentiate and display positives and negatives (including mixed positives) from each sample tested. The ddPCR was found to be simple and effective with small sample volumes. However, the authors indicated that the assay yielded a negative fluorescent signal when the plasmid concentration was too high, indicating that there may be an upper limit of detection [90]. This is important to note, as this could affect the results of samples taken from clinical animals experiencing peak shedding of a particular virus and thus result in a misdiagnosis. However, there is the possibility that this limitation might be able to be resolved through sample dilution. Additionally, it might be less of a concern with real clinical samples given the inhibitors and other materials which could be present in the samples. However, this limitation should be addressed in future developments of ddPCR assays and testing protocols to ensure that they are robust and able to yield reliable results for all potential clinical cases.

### 6.6. Isothermal Amplification Assays

While PCR and similar techniques offer high levels of sensitivity and specificity and have been greatly enhanced to provide quicker turnaround times and easier techniques, they do have some inherent drawbacks. They depend on varying thermal cycles for amplification of the target nucleic acid, and thus on sophisticated equipment that may not be suited to resource-limited situations. To overcome this, diagnostic assays have been developed that are independent of these thermal cycles and are thus termed isothermal amplification methods or assays. In essence, these assays make use of a stable temperature (which differs according to the procedure) for the full amplification process and use biochemical reactions and the action of primers and enzymes (particularly polymerases) to facilitate this. One of the most prevalent diagnostic assays following this approach is the loop-mediated isothermal amplification (LAMP) assay. Two other similar approaches that are based upon isothermal amplification that have been tested for BCoV are recombinase polymerase amplification (RPA) and multienzyme isothermal rapid amplification (MIRA), which is a derivative of single primer isothermal amplification.

RPA is an impressive development in the line of isothermal amplification assays, and due to the simplicity and rapid nature of these assays, they threaten to replace traditional PCR assays. This is largely due to the fact that tests can be run just above room temperature (no thermal denaturation), require few primers and enzymes, have a low test time, and provide high diagnostic sensitivity and specificity [63]. Zhang and colleagues (2024) published a thorough review highlighting the current state of RPA assays for the detection of a broad range of pathogens which is an excellent source for additional background on these assays [63]. In the review, the authors highlighted the underlying mechanisms and different detection methods related to isothermal amplification assays in general, particularly RPA, and also compared some of the relative advantages and disadvantages of different techniques.

A reverse transcription loop-mediated isothermal amplification (RT-LAMP) assay was designed by Qiao et al. in 2012 to detect BCoV [64]. The primers for the assay targeted the BCoV nucleocapsid (N) protein gene, and to aid detection and interpretation, the researchers made use of a SYBR Green 1 to easily visualize gross color changes in the tube, as well as gel electrophoresis. The sensitivity and specificity of the assay were very high, with a 98.2% agreement with a semi-nested RT-PCR on clinical samples [64]. The authors indicated how the sensitivity of the assay decreased when testing samples with feces, where initial testing of “samples” with serial dilutions of a mixture from plasmids expressing BCoV achieved detection with as low as 10 copies of plasmid DNA (without feces). When this was repeated where the plasmid was mixed with bovine feces, the assay showed a limit of detection of 10^2^ copies of the plasmid DNA [64], highlighting that this should be considered when testing the limits of detection for assays, especially based on synthetic models and if the model is applied between different sample mediums (e.g., feces vs. nasal secretions vs. blood).

The use of RT-LAMP was taken further by researchers in Germany who designed a duplex device aimed at diagnosing viral agents, particularly respiratory pathogens [65]. Their device could detect bovine coronavirus and influenza A/X-31 virus (they used this combination as a model for human respiratory infection that might include SARS-CoV-2), and the fully integrated system performed well for both pathogens [65]. The device was designed with all of the primers, reagents, and a lateral flow strip included and thus did not require additional specialized equipment, and it produced a color band to indicate a positive reaction. The total test time was reported as one hour, including the incubation time at 60–72 °C for the initial LAMP reaction. The limit of detection for BCoV using purified samples was 2000 RNA copies/mL, and this was even lower for influenza A. Unfortunately, however, this was not tested with feces or clinical samples [65]. Further considerations are that the assay in this state largely reports a qualitative result (positive or negative) with no quantification, and the authors did not report on the specificity and potential cross-reactions with other similar viruses beyond the two tested. With this said, this is very encouraging as to the possibilities that exist with portable, self-contained assays based on LAMP and similar technology.

Amer et al. (2013) reported the development of an assay using RT-RPA combined with fluorescence for the detection of BCoV based upon the nucleocapsid (N) gene that provided a very high level of sensitivity, apparently with a limit of detection of 19 RNA molecules in pure samples [66]. The authors reported a high specificity, as there were no cross-reactions with other viruses causing gastrointestinal and respiratory disease when tested. This assay promises high flexibility and ease as the reported run time is 10–20 min and the assay is performed at the isothermal temperature of 37–42 °C, which favors resource-limited setups [66]. Further development of portable RPA devices and additional clinical testing will likely enhance the widespread adoption of these assays in diagnostic testing.

Ji and colleagues (2023) developed another rapid detection device suited to a field setting based upon multienzyme isothermal rapid amplification (MIRA) and lateral flow dipstick [67]. Their assay targeted the conserved N gene (nucleocapsid) of bovine coronavirus and provided a result that could be read by the eyes without further equipment. The assay was seen to function best at 37 °C, which is certainly achievable in a field setting, and could detect as few as 100 copies of the plasmid following serial dilution tests [67]. This MIRA assay was tested against an RT-qPCR assay on clinical samples consisting of feces and nasal swabs. In this comparison, the MIRA LFD assay performed favorably, as the Kappa value from the samples tested was 0.982 [67]. However, it would be best to test a larger sample pool and to evaluate factors such as the positive and negative predictive values of the assay. This study demonstrates the potential feasibility and promising performance of this technology within assays for field or resource-limited conditions.

### 6.7. Microarray

DNA hybridization as a means of viral detection for BCoV was used by Verbeek and colleagues in 1989 through the use of an assay based on cDNA probes [91]. Their reported probes targeted the N and E1 (nucleocapsid and matrix proteins, respectively) genes, and they reported that their assay outperformed an ELISA in their tests, which included clinical fecal samples. This has since been taken a lot further by Chen et al. (2010) through the development of a multiplex microarray hybridization assay that could detect seven closely related animal coronaviruses, including BCoV, and human respiratory coronavirus (HRCoV) [92]. The researchers made use of cDNAs on specific gene chips, followed by PCR amplification, and after adjusting the cDNAs (primers), they had very high sensitivity and specificity and were able to distinguish all eight coronaviruses. The researchers reported that the assay was 1000 times more sensitive than the RT-PCR alone that was used for the validation. More recently, Thanthrige-Don et al. (2018) developed two multiplex PCR-electronic microarray assays for a total of twenty pathogens involved in bovine respiratory and enteric diseases [93]. The bovine respiratory disease multiplex array included nine pathogens (four bacteria and five viruses, including BCoV), while the bovine gastrointestinal disease multiplex assay included eleven pathogens (four bacteria, three protozoa, and four viruses, including BCoV). The electronic hybridization platform was able to identify and distinguish the various pathogens and to provide very high levels of sensitivity and specificity that rivaled PCRs used for testing and validation [93]. Unfortunately, the study only included a limited number of clinical samples for testing and validation, and while the results were good, this is something that should be expanded upon to see the performance of potential inhibitors and other factors that may influence the performance of the assay.

### 6.8. Enzyme Immunoassays

Historically, proteome-based BCoV detection methods such as direct/indirect ELISAs were quite common prior to the development and adoption of various sophisticated transcriptome-based pathogen detection tools.

Schoenthaler and Kapil (1999) were amongst the first to develop and report an ELISA test that was economical, rapid, and highly sensitive [56]. Their sandwich ELISA based on a monoclonal antibody for BCoV was tested on fecal samples and allowed detection of virus at 10^4^ particles in a sample, while electron microscopy only allowed detection at 10^5^ particles per sample [56]. The results from their developed ELISA found a close correlation and high specificity when samples were cross-tested with electron microscopy and an HA test. However, in this study the HA test performed poorly and had reduced sensitivity, likely resulting from the use of fecal samples, which highlights the importance of correlating diagnostic samples with the optimal assay [56].

Näslund et al. (2000) aimed to develop a robust ELISA for the detection of BCoV, where they used both serum and milk samples from Swedish cattle [57]. Fecal samples were not used due to observations of reduced sensitivity owing to low detection rates, presumably due to non-specific cross-reactions. The investigators developed and used a capture ELISA targeting IgA and IgM antibodies specific for BCoV, which was seen to perform well and seemed capable of discerning primary infection from reinfection [57]. This is favorable given that most animals tend to be exposed in early life or if a vaccination strategy existed, and thus this would avoid the necessity for paired serum samples. The researchers had tested potential indirect ELISAs originally, but these had reduced sensitivity and high levels of background reaction and were thus outperformed and replaced by the capture ELISA [57]. It was noted that the IgA and IgM antibodies were highly stable and tolerated freezing, thawing, and storage at room temperature, which would suit bulk and field sampling techniques. Additionally, milk presents a very convenient sample source from adult animals, particularly within a dairy herd structure, which further ties into bulk herd testing and surveillance.

More recently, researchers in Norway evaluated a multiplex immunoassay for BCoV and BRSV (bovine respiratory syncytial virus) against commercial indirect ELISAs (SVANOVIR BCV-Ab. SVANOVA, Uppsala, Sweden) against the respective agents to test for antibodies in bulk milk tank samples [58]. The multiplex assay made use of a panel of three recombinant proteins (A–C) as antigens, and the ELISA made use of an unspecified BCoV antigen to detect antibodies (IgG) in the bulk milk samples tested. With optimized conditions and through a Bayesian latent class model, focusing solely on BCoV, the researchers found the multiplex delivered a sensitivity of 99.9% and specificity of 93.7%, and the ELISA provided a sensitivity and specificity of 99.5% and 99.6%, respectively [58]. These results are astounding and certainly validate the use of these assays for herd screening with bulk milk samples, as was the case here, and one would assume that this would carry over when testing individual milk and serum samples. The BCoV-Ab ELISA tested (SVANOVIR BCV-Ab. SVANOVA, Uppsala, Sweden) is currently still available commercially, and according to the distributors, is used in many high-income countries across the world. This assay is primarily recommended for BCoV herd surveillance and control programs.

Immunochromatography tests, also called lateral flow tests or devices, are loosely based upon similar principles as ELISA assays and have posed an area of interest for further development. At their core, ICTs offer a portable and quick diagnostic assay that is not limited to specialized laboratory equipment or specific expertise, making them suited to field diagnostics. Researchers in Austria evaluated a commercial rapid ICT (FASTest BCV Strip) against the gold standard of RT-PCR using feces from calves [59]. The ICT was reported as providing a low sensitivity of 60% for BCoV detection, but a good specificity of 96.4%. The researchers also acknowledge the benefit of this as a rapid assay suitable for processing large numbers of samples and the potential use of pooling samples to increase sensitivity and suit a herd situation [59].

Reshová et al. (2001) developed an ELISA based on antigen detection using monoclonal antibodies and an ICT using polyclonal antibodies to BCoV. With the ELISA, two monoclonal antibodies against the S protein (formally called E2 outer structural protein) and two monoclonal antibodies against the N protein (inner capsid protein) were generated [60]. Monoclonal antibodies were cited as favorable over polyclonal antibodies alone, as the latter have low sensitivity and have been seen to be affected by non-specific cross-reactions including those with rotaviruses when testing fecal samples [60]. All four monoclonal antibodies were validated through testing by western blot, HA inhibition, and immunoperoxidase tests, where the virus was accurately identified, and additionally, no cross-reactivity to bovine rotavirus was detected. They developed and tested a sandwich ELISA using the monoclonal antibodies on fecal samples from calves and cross-validated this with electron microscopy. The authors determined good agreement (87.7%) between these assays for the samples tested, while also noting the increased ease of use of the ELISA [60]. The developed monoclonal antibodies were used in combination with polyclonal antibodies for BCoV to develop an immunochromatography test (ICT) that could be read within 10 min. The antibodies used in the developed ELISA and ICT were able to detect both intact and incomplete virions of BCoV, thus increasing the diagnostic sensitivity. The developed ICT was also tested on 74 calf fecal samples which were cross-checked with the previously developed ELISA as a reference, and the sensitivity of ICT was 94.9%, while the specificity was 86.7% [60]. The validity of the cited sensitivity and specificity should be considered after using the newly developed ELISA and in the absence of a comparison against a preferred assay such as qPCR; however, these results are promising for a rapid test.

## 7. Comparison of Assays in the Field

A practical, field-based study tested feces collected from calves in dairy herds in Australia that had outbreaks of diarrhea to compare ELISA, qRT-PCR, and a lateral flow immunochromatography test (ICT) to diagnose bovine coronavirus and rotavirus [16]. In this study, qRT-PCR was used as the gold standard for both assays, while the commercial ELISA also served as a reference for the ICT. The PCR primer was targeted at the ORF1ab gene of bovine coronavirus, while the ELISA kit (Pourquier^®^ ELISA Calves Diarrhea, Institut Pourquier^®^, Montpellier, France) made use of a polyclonal antibody against the BCoV spike (S) protein from the viral envelope [16]. The lateral flow assay was a commercial assay (Bio-X Diagnostics, Rochefort, Belgium), which does not appear to be available commercially any longer, and the specific BCoV antigen targeted by the antibodies in the assay was not noted. In their study, the in-house qRT-PCR, with a TaqMan probe, detected BCoV in 22.3% of the samples tested (131/586). The Ct values of the assay allowed for a degree of quantification of the viral load to assist with the interpretation of the results. With the PCR assay as a reference, the ELISA provided a poor sensitivity of 26.7% and specificity of 91.7%, while the positive predictive value (PPV) was 48% and the negative predictive value (NPV) was 81.3% in this population [16]. There was agreement (inverse correlation) between the ELISA S/P ratio and the qRT-PCR Ct values, both of which help quantify the degree of viral load. On the other hand, there was poor agreement between the BCoV lateral flow test compared to the PCR and ELISA tests. Compared to the PCR, the ICT provided a PPV of 36.7% and NPV of 72.6%, and compared to the ELISA, the PPV was 33.3% and 80.4% in the study [16]. These results from the tested ICT assay are disappointing and would likely preclude this assay from any use other than herd screening, with the main redeeming feature being the potential option for a cow-side test. On this note, the investigators did note that the results from the lateral flow dipsticks might have been better if the assay had been run at the point of collection and not collected and undergone transport for later testing in the laboratory. Regarding the ELISA test that was used, this also offered an element of quantification of viral load and allowed large numbers of samples to be tested readily and easily. This is a definitive advantage over conventional PCR assays. However, with the introduction of qPCR, these benefits are somewhat diluted as this modality also allows for quantification of viral load through Ct values (as mentioned) and modern testing procedures are more refined and simplified to facilitate rapid and accurate evaluation of large sample numbers.

More recently, researchers in Iran followed a very similar approach to compare ELISA, PCR, and an immunochromatography test to detect bovine coronavirus and rotavirus [61]. Both the ELISA and the ICT assay used were commercial multi-agent detection tests from the same manufacturer and these products are still commercially available, along with other similar tests with various agents. The ICT used was RAINBOW BIO K 288 (BIO-X Diagnostics, Belgium), which is a vertical flow immunochromatographic assay using a monoclonal antibody and colloidal gold-labeled antibody used for the detection of rotavirus, bovine coronavirus, cryptosporidium and the F5 attachment factor of *E. coli* [61]. The ELISA kit used was the BIO K 348 Multiscreen AgELISA (BIO-X Diagnostics, Belgium) which is a sandwich ELISA using monoclonal antibodies and is used for the same infectious agents as the BIO K 288. The manufacturer reports that the ELISA has a detection limit of 40,000 TCID_50_. In this study for BCoV, the ELISA was reported to provide a sensitivity and specificity of 100% and 98.53%, respectively, while the NPV and PPV were 100% and 95.7%, respectively [61]. Similarly, the ICT reported a sensitivity of 90.91%, a specificity of 91.18%, and the NPV was 96.9%, while the PPV was 76.9% [61]. These results indicate a significant improvement in the diagnostic performance of these assays compared to earlier studies and help to illustrate their value as potential options in a diagnostic setting.

Around 2012, Cho and colleagues investigated another ICT-based dipstick kit for the detection of four infectious agents causing diarrhea (bovine coronavirus, rotavirus A, *Escherichia coli* K99^+^, and *Cryptosporidium parvum*) compared to a multiplex real-time PCR assay [62]. The ICT was a commercial dipstick (Bovine Enterichek^®^ produced by Biovet Inc., Saint-Hyacinthe, Quebec, Canada) which appears to still be marketed, and is a multiplex assay able to provide a qualitative result for the four agents mentioned with a single test. The PCR was used as the gold standard, and serial dilution was used to ascertain a limit of detection of 300 copies/mL for the Enterichek (for all agents tested) [62]. While the ICT appeared to perform well for the other agents tested, for bovine coronavirus, the results in the study were disappointing, with a sensitivity of 60% and specificity of 51.4% compared to the manufacturer’s reported sensitivity of 63.6% and 97.4% for BCoV [62]. However, this still highlights the key advantage of immunochromatography-based assays, as they are capable of quickly providing workable diagnostic results for several agents within a field setting. This is particularly valuable for screening large numbers of animals, and where more precise results are required the test can be followed with a PCR, which was also supported in the study by Hamedian-Asl and colleagues (2022) [61]. However, as has been suggested, the further development of ICT-based assays and other point-of-care-tests (POCTs) has resulted in improved commercial assays that reduce the potential gap in results between them and validated assays and thus support their use in practice.

Interestingly, the same ELISA assay (SVANOVIR BCV-Ab. SVANOVA, Uppsala, Sweden) that was previously discussed was used by researchers in the Campania region of Southern Italy to evaluate the seroprevalence and risk factors of BCoV infection in cattle and water buffalo [94]. They expressed a seroprevalence of 5.3% in the water buffalo and 49.2% in the cattle that were tested, and unsurprisingly a risk factor for infection in the buffalo was cohabitation with the cattle [94]. Beyond this, however, is the fact that the researchers used the same ELISA assay for virus detection between the water buffalo and cattle, with distinctive results. In the absence of diagnostic assays validated for use in these other ruminant species, assays using antibodies developed for BCoV in cattle are being used in these species. Many of these assays using BCoV monoclonal antibodies appear to offer cross-reactivity to the BCoV-like viruses, as supported by HI, IFA, and neutralization assays [37,94]. Beyond highlighting additional potential use cases of these diagnostic assays (and the need for their validation for other species), this indicates that cross-reactions and false positive reactions may occur between these viruses in practice. Naturally, the degree of cross-reactivity will depend on the target of the assay and how conserved that site is between the viral variants in question.

## 8. Impact

The impact of BCoV within a herd is multifactorial but distinct. Considering the ubiquitous nature of the disease, the fact that it has a number of different forms, and that different age and production groups may be affected, it can have a profound impact on health, production, and welfare. It is hard to fully quantify the full economic impacts of the disease, especially given the difficulty in recreating infection with experimental and field challenges and considering that the virus is typically found in the presence of other pathogenic microbes. A study conducted within large dairy herds in Norway in 2017 demonstrated the profound effect that BCoV outbreaks had on the production of these animals and their milk production [45]. It was estimated that there was an average milk loss of 51 L per cow in the 26-day study period (7 days before and 19 days after the outbreak was reported). In affected herds, the average milk yield decreased to 19.4 L/cow/day, compared to 23.0 L/cow/day in unaffected herds, with the lowest daily yield seen two days post-reporting of the BCoV outbreak. The researchers further indicated a potential effect on milk quality in affected animals as well [45]. These results and the tremendous effects on milk production are similar to that described by another report where an outbreak of winter dysentery caused, amongst other things, a 30% drop in milk production in cows [44]. At a herd level, this loss of milk will have a tremendous effect on a producer, particularly when considering how quickly BCoV can spread in a herd and that the bulk of the herd is likely to be exposed to the virus in an intensive production system.

Furthermore, BCoV has tremendous impacts on health and production as a respiratory pathogen and agent within the bovine respiratory disease complex [3,29,95]. A study from 2020 evaluated the economic effects of bovine respiratory disease on animal performance and associated economic effects within a feedlot setting such as carcass quality and yield [96]. This robust study looked at various approaches to defining disease and affliction, including reviewing the potential impact of disease on subclinical carriers, which helps to provide a complete picture as well as a benchmark for comparisons. It is important to indicate that this study did not focus on a particular disease agent and thus cannot be ascribed to the impact of BCoV solely; however, as an agent causing BRDC, it is relevant for such comparisons. In their study, the impact of BRD morbidity was variable depending on how this was categorized. The greatest losses observed in the study, as expected, are attributed to mortalities, where there was an estimated average net loss of AUD 164,753 per mortality [96]. This cost included the purchase price as well as induction (processing), transport, feed, yardage, and treatment costs, as well as the cost of body removal. These are valuable considerations when quantifying the impact of a disease on a production system. One could, however, argue that the true loss could be taken further to consider the true economic costs of mortalities. In this regard, one may consider the potential slaughter value (price received) that might have been achieved while also estimating further costs for additional days on feed, etc., if this animal had survived to slaughter. Furthermore, if this was not a feedlot setting, one could consider other animal products, such as milk, as well as the potential offspring that may have been produced and the related loss of genetic potential. In the observed study, the surviving group with the greatest observed impact from infection was those animals treated more than three times for BRD which had 39.6 kg lighter carcasses at slaughter [96]. This was followed by the cattle with clinical BRD, which had 24.1 kg lighter carcasses than healthy and untreated animals without lesions [96]. It is noteworthy that cattle that were deemed as having subclinical infection with BRD were found to have notably reduced growth rates, with carcasses found to be 16.0 kg lighter than healthy animals that were never treated for BRD and had no associated lung lesions at slaughter [96]. This is an important finding as it demonstrates the effects respiratory infections can have, even in cases where there may be no obvious clinical signs or disease detection. In many studies, the potential role of subclinical infection is neglected, so we may not have a complete understanding of the true impact that these pathogens have on production. This reinforces the fact that we should seek to enhance diagnostics and husbandry practices such that we are able to detect infection in these subclinical cases more accurately.

## 9. Conclusions

Bovine coronavirus is endemic in the United States, Europe, South America, and Asia, and there are reports of epidemics in Africa and Oceania as well. Further studies on BCoV are recommended and warranted in order to investigate the full details that underpin viral transmission and tropism. It is important to understand these factors and the interplay of viral variants, potential host factors, pathogenesis and the mechanisms of disease, as well as the development of host immunity. Lastly, it is important to understand how these various factors affect and can improve diagnostics. This should ideally be taken further to help propose and develop enhanced prevention and control measures such as effective vaccines and vaccination strategies. Currently, diagnostics are largely based on PCRs, ELISAs, and ICTs, having largely replaced EM in most modern settings. It is likely that isothermal amplification assays will start to become more widespread as this technology is enhanced and more commercial assays are developed. It is believed that POCTs have a significant and important role to play in diagnostics for BCoV, and while some assays have been made available in the last decade, additional investment and development in these assays will be of tremendous value. It is believed that POCTs will likely become the standard diagnostic assays for livestock diseases such as BCoV (and other BRDC agents) and that further laboratory diagnostics will be reserved for limited cases or where further investigation or validation is required.

## Figures and Tables

**Table 1:** Diagnostic tests used in the detection of bovine coronavirus.

	PCR	ELISA	Isothermal Amplification Assays (LAMP, RPA, MIRA)	Sanger Sequencing and Next-Generation Sequencing	Virus Identification (Immuno-Fluorescence Assays and EM)
Target	Nucleic acids (viral RNA). Specific gene target such as N and S gene.	Antibody (IgA, IgG, IgM) or antigen detection (structural proteins such as E2 or S).	Nucleic acid (viral RNA). Specific gene targets such as the N gene.	Nucleic acid (viral RNA). Most commonly used for segments of genome or whole genome.	EM: Virus particles. Immunofluorescence: Virus particles or components.
Samples	Almost any sample with viral nuclear material (RNA)—commonly nasal/conjunctival/rectal swabs, feces, and tissues (respiratory and intestinal) at necropsy.	Sserum, plasma, and milk (colostrum). Feces for antigen test.	Almost any sample with viral nuclear material (RNA), as with PCR. Commonly nasal/conjunctival/rectal swabs, feces, and tissues (respiratory and intestinal) at necropsy.	Sample with high-quality viral nuclear material (RNA), e.g., serum.	Largely tissue samples or virus isolations. EM: Nasal/rectal swabs and feces can be used with adequate processing steps.
Mechanism	Amplification of nuclear material based on targeting specific segments using heat cycles and reagents. qPCR (real-time) is most common. Multiple variations such as nested, semi-nested, and multiplex PCRS.	Both direct and indirect ELISAs are available. Makes use of specific antigens or antibodies (often monoclonal) to target and bind specific antibodies or specific sites on antigens, respectively. Reporters are used to indicate binding (positives) through various mechanisms.	Amplification of nuclear material targeting specific gene segments using isothermal reaction and multiple primers and reagents. Reactions largely driven by enzymes and primers.	Can identify full genome sequence from available nuclear material. tNGS- targets and amplifies a specific segment to aid detection.	EM: Physical identification of virus particles. Immunofluorescence assays: antibody binding and fluorescence to detect viral presence.
Advantages	Very high sensitivity and specificity (gold-standard assay). Rapid results and highly accurate with validated systems. qPCR allows for real-time analysis and some quantification of viral load. Multiplex and panels help with disease syndromes.	High (to very high) sensitivity and specificity. Rapid and well-suited to large sample numbers (herd tests). Commercial kits are available, and some combine lateral flow tests for rapid results. Allows degree of quantification. Can be used to detect antibodies and antigen.	Very high sensitivity and specificity. Rapid results and highly accurate. Can be combined with rapid readout systems (such as lateral flow strips). Less need for laboratory equipment. Multiplex is possible.	Very high sensitivity and specificity. Can detect multiple pathogens at once (multiplex) with fewer limitations. Can characterize viruses and help identify new virus variations (outbreak situation).	Immunostaining can be compared with pathology to support causation instead of presence alone. Specificity is moderate to good for immunostaining.
Disadvantages	Multiplex assays are limited in the number of pathogens that can be included. Laboratory based.	Care must be taken with interpretation as antibodies may not represent current infection. Confirmation with PCR may be warranted in some cases. Sensitivity and specificity are not as high as molecular tests.	Systems are still in development to provide a complete assay that can be widely adopted and integrated into practice.	Not widely used in diagnostics currently—slower and more expensive than other assays. Requires good and high-quality nucleic acid.	Direct detection depends on correct sample collection and processing to facilitate diagnosis. Time-consuming and costly. Sensitivity can be low-moderate depending on sample and assay. Specificity is moderate for a species level—depending on sample and assay.
Other	Considered the gold standard. Most widely used assay. Presence of antigen does not necessarily correlate with disease, thus quantification is helpful.	Rapid commercial tests are being improved to provide increasingly quicker and more reliable assays.	Can also be used with probes (such as TaqMan) to allow real-time detection.	Currently better suited to population studies and outbreak investigations. Further developments in technology may make this more widespread.	Largely replaced by molecular mechanisms. Immunostaining is mostly used for necropsy and tissue samples. EM: May only be specific to the family level. Limited application in modern practice.
References	[16,17,47,48,49,50,51,52,53,54,55]	[16,56,57,58,59,60,61,62]	[63,64,65,66,67]	[68]	[4,19,29]
